# Metastatic Cardiac Hemangiosarcoma in a 6 Year Old Wheaten Terrier Mix

**DOI:** 10.3390/vetsci6030065

**Published:** 2019-07-23

**Authors:** Shiori Arai, Ellen P Milley, Jonathan Lichtenberger, Christine Savidge, Jessica Lawrence, Etienne Côté

**Affiliations:** 1 Department of Companion Animals, Atlantic Veterinary Collage University of Prince Edward Island, Charlottetown, PE C1A 4P3, Canada; 2 Veterinary Clinical Sciences Department, College of Veterinary Medicine, University of Minnesota, Minneapolis, MN 55108, USA

**Keywords:** cardiac hemangiosarcoma, metastasis, *Coriolus versicolor*, metronomic chemotherapy

## Abstract

A 6 year old Wheaten Terrier mix with a history of collapse and lethargy was referred for evaluation of pericardial effusion. The echocardiogram identified pericardial effusion and a right auricular mass. No sign of metastasis was noted at this time in thoracic radiographs and abdominal ultrasounds. The patient underwent the right auriculectomy via right lateral thoracotomy. Several metastatic masses were located in the visceral aspect of the pericardium at the time of surgery and were all excised. The right auricular mass and pericardial masses were diagnosed as hemangiosarcoma with a sign of metastasis. The patient recovered from surgery uneventfully and was discharged the sixth day after surgery. The patient received doxorubicin followed by cyclophosphamide, piroxicam and *Coriolus versicolor* extract postoperatively. Pulmonary metastases were noted 229 days and the dog was euthanized 318 days after surgery. No clinical signs were noted until 309 days postoperatively.

## 1. Introduction

Hemangiosarcoma is an aggressive malignant tumor originating from the vascular endothelium and is commonly seen in the spleen, right auricle and liver. It is the most common type of malignant cardiac tumor in dogs, accounting for approximately 70% of cardiac neoplasms in dogs [1,2]. Although cardiac tumors can be seen in any area within the heart (i.e., in the left ventricular free wall, in the interventricular septum or both) [3,4,5], the majority of primary cardiac hemangiosarcomas involve the right atrium or auricle [2,6,7,8,9]. Due to its highly invasive nature, the prognosis of cardiac hemangiosarcoma is reported to be poor with a reported median survival time (MST) of 7 days in non-treated dogs [8], between 42 days and 5 months in dogs with surgical removal of the tumor only [8,10,11], 139 days in dogs with chemotherapy only [12] and between 175 days and 189 days in dogs with surgical removal of the tumor and conventional chemotherapy [8,11].

The present case describes the clinical presentation, surgical treatment and long-term postoperative management consisting of traditional and metronomic adjunctive chemotherapy plus *Coriolus versicolor* extract administration in a dog with metastatic cardiac hemangiosarcoma.

## 2. Case Presentation

A 6 year old, 33 kg female spayed Wheaten Terrier mix presented to a local veterinary clinic due to severe lethargy and weakness after vomiting a large amount of clear fluid. No prior health issues were reported; however, the dog had been less energetic for a week prior to presentation, according to the owner. The physical examination at that time identified tachycardia and muffled heart sounds during thoracic auscultation. Thoracic radiographs revealed an enlarged cardiac silhouette and an ultrasound examination showed pericardial effusion. Pericardiocentesis was performed and 110 mL of hemorrhagic pericardial fluid was removed. The patient received intravenous fluid therapy and was referred to the small animal hospital at the Atlantic Veterinary College (AVC) for further evaluation.

On initial evaluation at the AVC, the patient was lethargic but ambulatory. Physical examination revealed muffled heart sounds, mild tachycardia and weak femoral pulses. The dog was slightly dyspneic but not in distress. No accurate systolic blood pressure measurement could be obtained on initial presentation using Doppler ultrasonography, likely due to peripheral vasoconstriction and/or hypotension. A complete two-dimensional, M-mode and Doppler echocardiogram revealed moderate anechoic pericardial effusion and a broad-based, 3.3 × 4.6 cm mass of mixed echogenicity involving most of the lateral wall of the right auricle. Diastolic collapse of the right atrium was present, consistent with cardiac tamponade. M-mode measurements of the left ventricle obtained from the right-parasternal short axis view were most consistent with concentric pseudohypertrophy secondary to cardiac tamponade and ventricular underloading. No evidence of distant metastasis was noted in three-view thoracic radiographs and abdominal ultrasound examination. Due to recurrence of pericardial effusion and the location of the right auricular mass, the owner was informed of treatment options and attendant advantages and drawbacks; the owner requested surgical excision of the mass.

A right lateral thoracotomy was performed in routine manner through the fourth intercostal space with the patient under general anesthesia. The pericardium was incised along its craniocaudal aspect ventral to the right phrenic nerve and a pericardial basket was created by suturing it to the thoracotomy incision. A 4 cm diameter mass was palpated in the right auricle and Satinsky vascular forceps were placed as close to the right atrium as possible to isolate the right auricle with the widest possible margin. The mass was excised and the auriculectomy incision was closed with three mattress sutures with pledgets using 4-0 polypropylene over-sewn with a simple continuous pattern using 3-0 polypropylene. Five small dark round masses ranging from 5 mm to 12 mm in diameter were adhered to the visceral surface of the parietal pericardium and were suspected to represent locoregional metastasis. They were carefully dissected with tenotomy scissors after the owner was notified of their presence and again provided consent. No other lesions were seen in the other areas of the pericardium. The pericardium was loosely apposed and sutured in a simple interrupted suture pattern using 3-0 polydioxanone. The right thoracotomy was closed in a routine fashion after a 24 Fr thoracic tube had been placed through the seventh intercostal space. Recovery from anesthesia was uneventful. The dog was hospitalized in the intensive care unit with analgesia (Fentanyl IV CRI 1–3 µg/kg/h, tramadol 3 mg/kg orally every 12 h, and meloxicam 0.1 mg/kg orally every 24 h) and supportive care. The dog experienced mild aspiration pneumonia on the fifth day postoperatively and was discharged with oral antibiotics (amoxicillin/clavulanic acid, 13.75 mg/kg every 12 h) 7 days after surgery.

**Figure 1:**
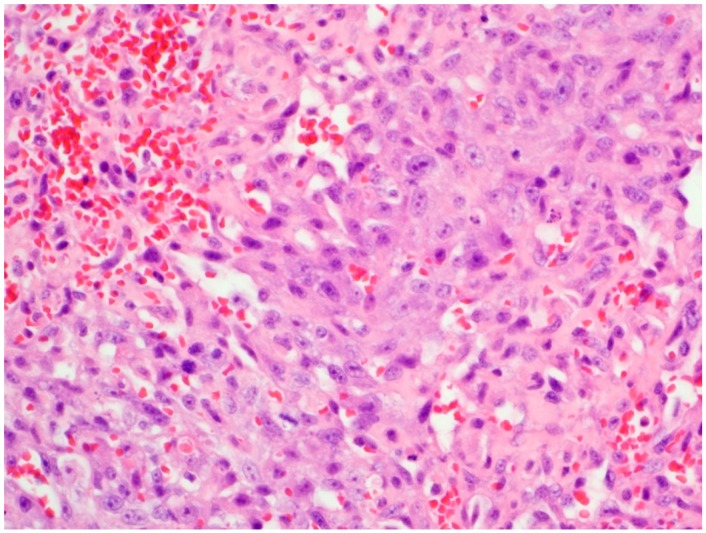
Histopathology of the pericardial mass.

Histopathological examination of the right auricular mass revealed locally invasive infiltrates of plump spindloid cells which formed small blood-filled channels and/or coalescing sieve-like clefts supported by small amounts of a fine collagenous stroma. These infiltrates multifocally breached the wall of the atrium and invaded the adjacent pericardial fat. The tumor cells had round to oval nuclei with finely stippled chromatin, often with a prominent nucleolus, and had small amounts of poorly defined cytoplasm. These findings were consistent with hemangiosarcoma. Anisokaryosis was moderate. Mitotic figures were 1–2 per high power field. Neoplastic infiltrates extended to all tissue margins. Sections of the pericardial masses also consisted of large areas of hemorrhage, occasionally interspersed with variably sized infiltrates of the tumor cells seen in the right auricular mass (Figure 1).

Fifteen days postoperatively, intravenous injectable chemotherapy was initiated with doxorubicin 30 mg/m^2^. This protocol was repeated every 3 weeks for five treatments with repeat staging consisting of a complete blood count and serum biochemistry profile along with three-view thoracic radiographs, a complete abdominal ultrasound and echocardiographic evaluations at the time of the third doxorubicin treatment. No sign of metastasis was noted in the thoracic radiographs, abdominal ultrasound examination, or echocardiogram. The dog experienced grade 3 gastrointestinal adverse events [13] 3 days after the first treatment, which resolved with supportive care without the need for hospitalization. Maropitant (Cerenia, Pfizer Animal Healthm Parsipany-Troy Hills, NJ; 1 mg/kg subcutaneously once followed by 2 mg/kg orally every 24 h for 4 days) was administered with each doxorubicin administration and the dog tolerated all remaining treatments without notable signs of toxicosis. After five treatments with intravenous doxorubicin (118 days postoperatively), metronomic chemotherapy was initiated with piroxicam (Novo-Piroxicam, Novopharm Ltd. Stouffville, Whitchurch-Stouffville, Canada; 0.3 mg/kg orally every 24 h) and cyclophosphamide (Procytox; Baxter Corporation, Mississauga, Canada; 20 mg/m^2^ orally every 24 h). Concurrent treatment with a polysaccharopeptide bioactive agent from the mushroom *Coriolus versicolor*^a^ (I’m Yunity^®^, Integrated Chinese Medicine Holdings, Ltd. HongKong, China, 100 mg/kg orally every 24 h) was administered starting 21 days postoperatively and continued lifelong. No abnormalities were noted in physical examinations and imaging diagnostics until the 229th postoperative day, at which time multiple soft tissue opacities were visible on thoracic radiographs, representing suspected pulmonary metastases.

**Figure 2:**
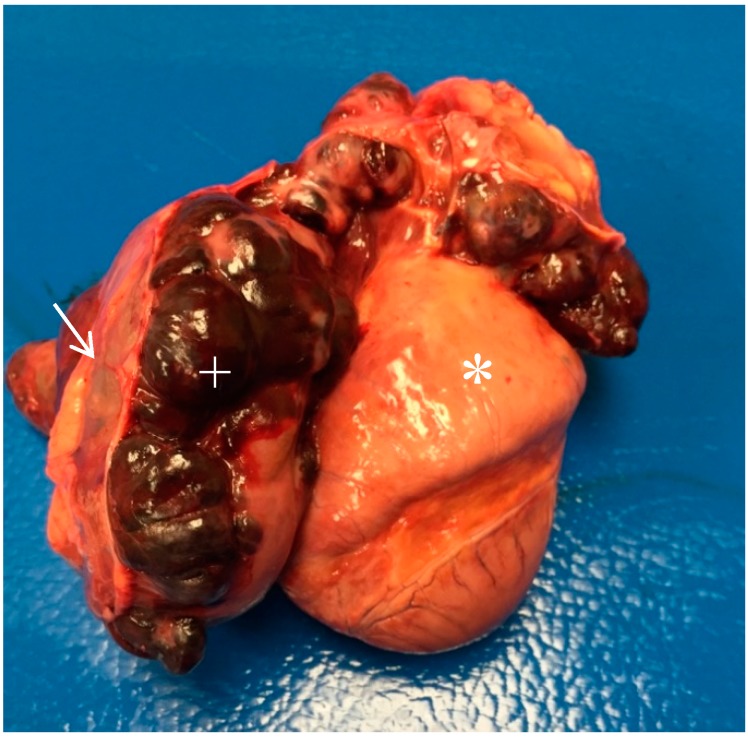
Gross picture of the heart (*) encapsulated by the tumor (+) under the pericardium (arrow).

The dog presented to the AVC emergency service with a week’s history of lethargy and vomiting, as well as hematochezia for 2 days prior to presentation (318 days postoperatively). The dog was tachypneic, dyspneic, and recumbent on presentation. Progressive pulmonary nodules were radiographically present. Given the likelihood of progressive metastatic malignant neoplasia despite treatment, the owner elected humane euthanasia. A complete necropsy was declined by the owner, but the heart and a part of the lungs were examined grossly with the owner’s permission. The heart and lungs showed multiple dark red, firm, infiltrated nodular masses ranging in size from 1 mm to 3 cm. The pericardial sac was distended with serosanguineous fluid and a large, 12–15 cm, firm, red, multi-lobulated mass extended from the heart base involving the right atrium and pericardium (Figure 2).

## 3. Discussion

Hemangiosarcoma (HSA) is an aggressive malignant cardiac tumor that is generally associated with a poor prognosis [1,9,11]. Surgical intervention (i.e., pericardiectomy with or without tumor removal) is performed for the purposes of diagnosis and prevention of cardiac tamponade rather than for curative intent or prolonged survival time because the median survival time is short (typically 5–6 months), even with surgical resection of the tumor followed by adjunctive chemotherapy [8,11]. If no treatment is performed, the survival time is even shorter (median, 7 days; range, 1–26 days) [8]. The longest survival time in a dog with cardiac hemangiosarcoma with no evidence of metastasis reported in the published literature in English was 251 days or 270 days after surgery with or without chemotherapy, respectively [14,15]. Although the disease processes and the treatment protocols used in these previous reports in dogs with cardiac hemangiosarcoma cannot be directly compared to the case presented in this study, it is noteworthy that the dog in this report survived 318 days postoperatively with evidence of pericardial metastasis at the time of surgery.

The locations of the metastases in dogs with cardiac HSA have been reported to be in the lungs, liver, other parts of the heart, subcutaneous tissues, adrenal gland, kidney, omentum, intestine and pericardium [8,10]. Reports of metastasectomy in humans have consistently shown that fewer metastases and a longer interval before detection of metastasis are associated with a longer survival time after metastasectomy in any tumor type [16,17]. Although information regarding the efficacy of metastasectomy in small animals is sparse, metastasectomy has been reported in a study of dogs with osteosarcoma that had distant lung metastasis [18]. This study showed that the disease-free interval was correlated with survival time, which is similar to the findings in humans—the removal of metastatic lesions in addition to the primary lesion was associated with longer survival times [19,20]. In addition, the number of metastatic lung nodules was a significant negative prognostic factor [18]. A study by Frankot and Behrend reported the long-term management of a dog with incomplete excision of an adrenocortical carcinoma that underwent metastasectomy of the liver lobes [21]. Successful long-term management was possible after the metastasectomy alone and the dog survived more than 3 years after surgery. These studies are not directly comparative to the present case because of the different disease process; however, the idea of metastasectomy for cardiac hemangiosarcoma should not be underestimated because it is possible that cytoreduction with metastasectomy may prolong the overall survival time in canine cardiac hemangiosarcoma, like the case in this report. A recent paper from Turner et al. also supported the notion that metastasectomy prolongs survival in dogs with osteosarcoma [22]. The mechanisms of loco-regional metastasis in canine cardiac hemangiosarcoma are not fully understood, as it may be due to hematogenous spread or transpericardial implantation. It is also unknown whether it indicates a worse prognosis. Further studies involving a larger number of cases are warranted to evaluate the true incidence of loco-regional metastasis and the subsequent significance of metastasectomy in canine cardiac hemangiosarcoma.

Metronomic chemotherapy is a newer approach to cancer patients in veterinary medicine. Metronomic chemotherapy involves lower dosages of chemotherapy drugs that target tumor vasculature instead of neoplastic cells to slow tumor growth by inhibiting angiogenesis [23]. In humans, it has been introduced as an alternative therapy for primary systemic therapy, especially for a patient that has a recurrence of cancer or metastatic or locally advanced neoplasm [24]. In this regimen, a chemotherapeutic drug is chronically administered frequently at low dosages over a long time period without prolonged drug-free periods. Its intent is to disrupt neoangiogenesis and inhibit immune suppression by the tumor rather than to target tumor cells [23]. Metronomic chemotherapy has been used in dogs with splenic hemangiosarcoma and has shown longer median overall survival time for dogs compared in dogs treated with traditional chemotherapy with doxorubicin alone (178 versus 133 days) [25]. Recently, a study in dogs with non-cardiac hemangiosarcoma showed a significantly improved survival time and time to metastasis when metronomic therapy was followed by the traditional chemotherapy regimen [26]. The authors in this study described that the metronomic therapy may enhance the immune system and thus activate or restore the antitumor properties through the inhibition of regulatory T-lymphocytes, which stimulates the cytotoxic T-lymphocyte response. Moreover, this approach may introduce potent anti-angiogenic properties, both of which may improve the outcome with continuous use. Contrary to these data, more recent studies in dogs with splenic hemangiosarcoma did not show any improvement in survival time when metronomic chemotherapy was added or substituted for the doxorubicin treatment [27,28]. A study reported by Mullin et al. presented cases with suspected cardiac hemangiosarcoma that received metronomic therapy. In this study, no survival benefit was seen, although certain cases did not undergo surgical removal of the tumor prior to metronomic therapy. The specific benefits of metronomic therapy for canine cardiac hemangiosarcoma are unknown. It is possible that the efficacy of metronomic therapy depends on the timing of the therapy, origin of the tumor (splenic versus cardiac) or the presence of gross tumor. It would be worthwhile to investigate its effects on prognosis in canine cardiac HSA, knowing that it was well tolerated in the long-term in the present case. The metronomic therapy used in the present report was well tolerated in the patient and no sign attributable to adverse effects was noted by the owner or veterinarians involved in case management, other than vomiting that responded to maropitant co-treatment during doxorubicin administration.

The patient in the present report received a mushroom extract^a^ as part of the adjuvant therapy following surgery. This substance is a compound derived from *Coriolus versicolor* (CV), a polypore mushroom used as a part of herbal medicine which is sold as I’m Yunity^®^ for veterinary patients. The polysaccharide peptide isolated from CV is the active ingredient and it has shown potential in cancer treatment with immune-modulating properties such as the reduction of cell proliferation rate, induction of apoptosis, decrease of the survival rate of neoplastic cells, and suppression of DNA synthesis [29,30,31]. Recently, a novel glucan extracted from CV was shown to decrease the proliferation rate of S-180 sarcoma cells and the weight of experimentally developed S-180 sarcoma tumors in mice [32]. Clinically, CV has been associated with prolonged survival rate in human cancer patients [33,34]. In a recent double-blinded randomized multi-dose study, this mushroom extract was associated with increased survival time in dogs with splenic hemangiosarcoma following splenectomy [35]. Although the number of patients in that study was limited (15 in total, five in each dosage group), patients supplemented at 100 mg/kg/day had a significantly greater MST than did the dogs given 25 mg/kg/day (199 versus 30 days). The supplementation with high-dosage polysaccharopeptide (100 mg/kg/day) was thought to delay the onset or progression of metastases. In the present case, no adverse effect was noted with this mushroom extract. The association between this supplement and the longer survival time in the present dog is unclear; however, the influence of polysaccharopeptide in dogs with hemangiosarcoma or other types of neoplasia is worth further investigation.

The dog described herein provides a good example of our inability to predict outcome despite a presumptively poor prognosis with multifocal metastasis at the time of diagnosis. It provides further support for the role of surgery in the long-term management of dogs with cardiac hemangiosarcoma, even for those with presumptive metastasis identified at surgery. It is possible that cytoreductive surgery may have a significant role in controlling the disease process and/or the multimodal approach, including metronomic therapy, which allows a longer survival time. This case also complements a recent case report [36] that documented long-term survival in a dog diagnosed with hemangiosarcoma in the eye and in the spleen, and that was treated with surgery and *Coriolus versicolor* extract as part of a multimodal approach. Randomized controlled prospective trials are ideal and essential to truly characterize the benefit of alternative approaches like *Coriolus versicolor* extract combined with traditional chemotherapy.
